# Intensive care unit outcomes in patients with hematological malignancy

**DOI:** 10.1097/BS9.0000000000000038

**Published:** 2020-01-16

**Authors:** Jarrod Leigh Rawson, Fionnuala Mary Fagan, Georgin Claire Burrough, Helen Michelle Tang, Moire Alys Cuncannon, Katrina Louise Ellem, Anoop Kumar Enjeti

**Affiliations:** a Hematology Unit, Calvary Mater Newcastle, NSW; b NSW Health Pathology North-Hunter; c Intensive Care Unit, Calvary Mater Newcastle, NSW; d University of Newcastle, Callaghan, NSW; e Hunter Medical Research Institute, New Lambton, NSW; f Hunter Cancer Research Alliance, NSW

**Keywords:** Comorbidity, Hematologic malignancy, Intensive care, Prognosis

## Abstract

Hematological malignancies are usually life-limiting conditions. Limitations of care need to be decided early, based on acceptability to the patient, family, physician, and community. Inappropriate intensive care unit (ICU) admission is likely to result in significant physical, psychological, and economic burden. There is little published on the impact of non-acute preadmission disease factors on ICU outcomes in hematological malignancies.

**Aim:**

To identify baseline performance and disease-associated factors before admission to ICU in patients with hematological malignancy that contribute to subsequent ICU mortality.

**Methods:**

A retrospective analysis of electronic medical records, laboratory results, and Intensive Care data for all patients (*n* = 184) with hematological malignancy admitted to the Calvary Mater Hospital ICU between January 1, 2013 and June 30, 2017 was undertaken. Baseline age, gender, condition, Eastern Cooperative Oncology, and Charlson Comorbidity scores were compared to ICU outcome and overall survival. Disease-specific prognostic risk scores were compared to ICU outcome.

**Results:**

Overall, 73.9% survived the ICU admission, with 31.6% surviving at 12 months. Superior ejection fractions (>55%) and prognosis >12 months (based on disease-specific risk scores) were significantly associated with overall survival (*P* = 0.024 and *P* = 0.001). Induction and posttransplantation therapy were predictive of poor ICU survival outcome (*P* < 0.0001 and *P* = 0.041). APACHE scores were significant predictors of ICU mortality (*P* = 0.002 for APACHE II and *P* < 0.0001 for APACHE III).

**Conclusion:**

Survival outcomes for patients with hematological malignancy admitted to the ICU correlate with functional and comorbidity status. Disease-specific prognostic scores can assist in recognizing patients likely to benefit from ICU admission.

## 1 INTRODUCTION

Patients with hematological malignancies (HMs) who are admitted to the intensive care unit (ICU), as a result of either the underlying condition or its treatment, have a high mortality risk.^1,2^ A diagnosis of HM alone is no longer an absolute contraindication for ICU admission.^3^ Consequently, increasing numbers of patients with life-limiting malignancies but receiving life-prolonging therapies are admitted for life-saving or sustaining treatments in the ICU.^4^

While historically prognosis of HM patients admitted to ICU has been regarded as poor, these outcomes have now improved and moved closer to the general ICU population.^1,2,5–7^ This is due to improved supportive therapies for critical illness, better management of chemotherapy-induced toxicities, and knowledge of malignancy-specific risk predictors.^8^ As a result, broader admission policies have evolved for patients with HM admitted to the modern ICU.

The well-established prognostic factors for ICU survival are usually specific to the underlying critical illness rather than risk factors relating to HM. These often inform the requirement for ICU admission, the likely course of the admission, and which interventions to offer.^9^ Multi-organ failure on admission is the most powerful risk factor, with increasing number of systems failing associated with a stepwise increase in mortality rates.^9–11^

Whether disease-related prognostic scores correlate with prognosis of HM patients admitted to ICU is not widely studied. A prognosis of less than 6 months or a condition with no life-span-prolonging treatment options is unlikely to benefit from ICU admission.^4^ However, some specific malignancy groups, including diffuse large B-cell lymphoma and subtypes of acute myeloid leukemia (AML), show favorable long-term prognosis if they survive the critical illness in ICU.^4,9,12,13^ Furthermore, the time course of the disease, such as newly diagnosed or relapsed, is likely a risk factor for higher ICU mortality.^9^ A previous analysis by Massion et al (2002) suggest that HM may determine long-term survival but not ICU or hospital survival.^14^ Well-validated prognostic markers such as disease-specific risk scores and cytogenetic markers can predict survival from HM, but their applicability to ICU survival has not been explored.^12,13^

Therefore, the aim of our study was to evaluate non-acute illness factors that may influence ICU survival outcome. Specifically, we studied in a regional (Australian) context measures of hematological illness (including disease-specific and cytogenetic risks), performance status, and comorbidity to assess or confirm whether they are useful in predicting ICU mortality for patients with hematological illnesses.

## 2 METHOD

A retrospective cohort study of all consecutive patients with a HM that were nonelectively admitted to the ICU of the Calvary Mater Hospital, Newcastle, between January 2013 and July 2017 was undertaken. The Calvary Mater Newcastle is the major hematology referral center of the Hunter New England Local Health District of New South Wales, Australia. It is a 195-bed public hospital, specializing in hematology, oncology, toxicology, and mental health, with a six-bed general ICU. The hospital cares for patients with a broad range of HM and performs autologous, but not allogeneic, stem cell transplants.

Data were retrieved via accessing the patients’ electronic medical records by a team of researching physicians and recorded in a confidential database. Variables were obtained from electronic medical records or calculated by standardized tools. Discrepancies were resolved by repeat verification of the record and/or discussion with a senior member of the team.

Information regarding demographics, hematological diagnosis, pre-ICU admission characteristics such as comorbidities, and performance status and outcome was collected. The time course of treatment, including whether chemotherapy was prescribed, and the phase such as induction, ongoing or consolidation, post-autologous or allogeneic transplantation, or palliative chemotherapy, was also recorded. Additionally, the intention of this treatment, being curative or palliative, was documented.

A senior hematology registrar retrospectively determined the hematological prognosis of the main groups of patients based on recognized prognosticating frameworks. The Follicular Lymphoma International Prognostic Index (FLIPI) was used to prognosticate follicular lymphoma; revised International Prognostic Index (R-IPI) for diffuse large B-cell lymphoma (DLBCL); the International Staging Score (ISS) for multiple myeloma; and the International Prognostic Staging Score (IPSS) for myelodysplastic syndrome (MDS). Leukemias were staged based on cytogenetic profiling into good, intermediate, and poor risk categories. The former two were classed as predicted survival greater than 1 year.^15,16^

Using the Eastern Cooperative Oncology Group (ECOG) Score, a value for individual performance status was assigned, at the time of admission and 3 months prior. A score using the Charlson Comorbidity Index (CCI) was measured at the time of admission. These scoring systems were chosen based on their wide familiarity, repeatability, and validation in hemato-oncology and ICU teams. Where available, ejection fraction by transthoracic echocardiography or nuclear medicine (less than or greater than 55%) was also obtained.

Data submitted to the Australia and New Zealand Intensive Care Society (ANZICS) Adult Patient Database were accessed to record Acute Physiology and Chronic Health (APACHE) II and III scores for the first 24 hours of ICU admission. A higher score is an indicator of the severity of disease. The major interventions performed in ICU were recorded (including invasive or noninvasive ventilation, vasopressors or inotropes, and hemodialysis). Survival from ICU, length of admission, survival of hospital admission, and 12-month survival (where available) were determined.

Statistical analysis was performed using IBM SPSS (Version 24, Armonk, NY). Each variable (age, gender, hematological prognosis, and ECOG and CCI scores) was compared to ICU outcome using univariate analysis. A further multivariate analysis was performed for the combined predictive effect of these variables on the outcome of ICU admission survival (as a binary variable) as well as overall survival (in months) for a total follow-up period of 12 months from initial ICU admission.

Approval for the study was obtained from the local area health ethics committee. Informed consent for the study was waived as this was a retrospective design, there was no intervention, and all data were treated securely and confidentially.

## 3 RESULTS

### 3.1 Demographics

**Figure 1:**
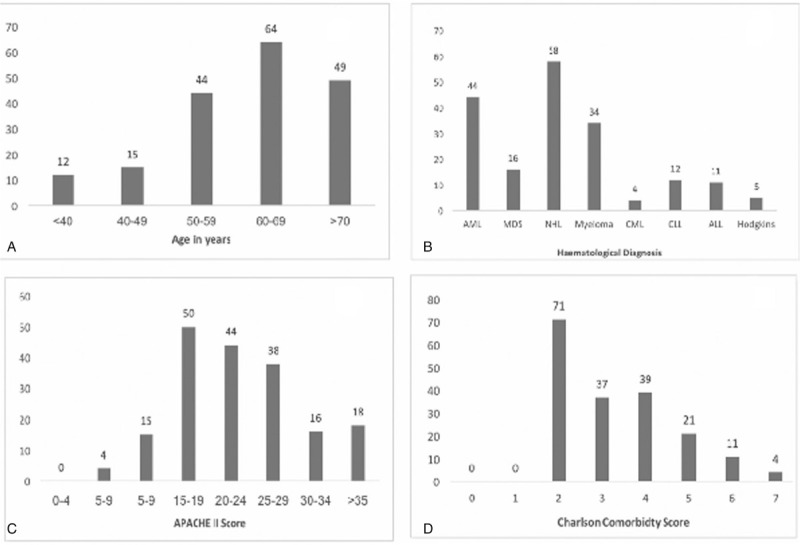
A) Age contributions. B) Breakdown of diagnoses. C) APACHE II scores.^23^ D) Charlson Comorbidity scores.

There were 184 admissions for patients with HMs out of a total of 2075 ICU admissions in the study period. There were more males (*n* = 127, 69%) and the mean age was 61.9 years (range 19–88 years). Figure 1A outlines the age contributions showing a preponderance of patients above 50 years of age. The most common diagnoses were non-Hodgkin's lymphoma (NHL) (*n* = 58, 31.5%), AML (*n* = 44, 23.9%), and multiple myeloma (*n* = 34, 18.4%) comprising three quarters of all patients. Figure 1B outlines the breakdown of the diagnoses.

### 3.2 ICU data

A total of 136 patients (73.9%) survived the ICU admission; 119 (87%) of ICU survivors also survived the hospital admission. The average length of stay in ICU was 4.4 days (±5.3). The mean survival for those who survived ICU admission was 6.5 months (±5), with 43 (31.6%) surviving for 12 months. The average APACHE II score was 23 (±40.3) with a range of 7 to 58. The breakdown of the APACHE II scores of the population is outlined in Figure 1C. Of ICU interventions, 102 patients received vasopressor/inotropic support, 52 were mechanically ventilated, 72 received noninvasive ventilation, and 13 had renal replacement therapy. Ejection fraction data were available for 112 patients (60.8%), with 88 of these having a known ejection fraction greater than 55%.

### 3.3 Prognostic factors

**Figure 2:**
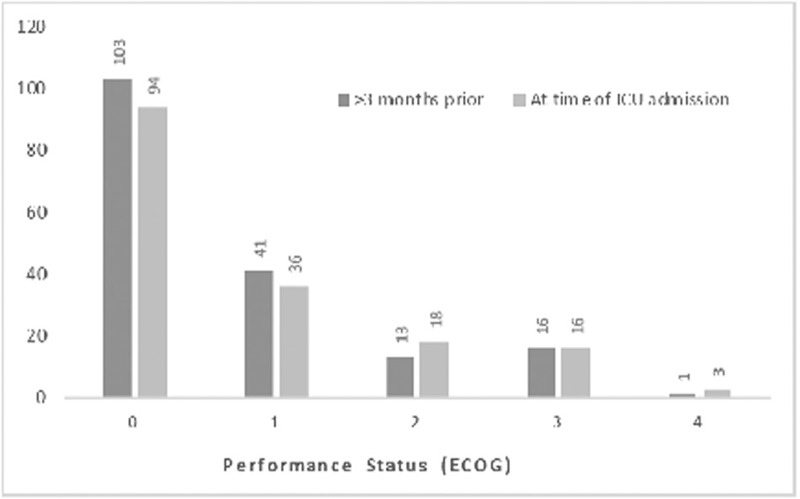
ECOG scores.

The majority of CCI scores were within 2 to 4, with a minimum possible score of 2 due to HM included in the CCI criteria, as shown in Figure 1D. As demonstrated in Figure 2, most patients had an unrestricted functional status, illustrated by the predominance of an ECOG score of 0. There was little change of this score in the months prior to ICU admission.

**Figure 3:**
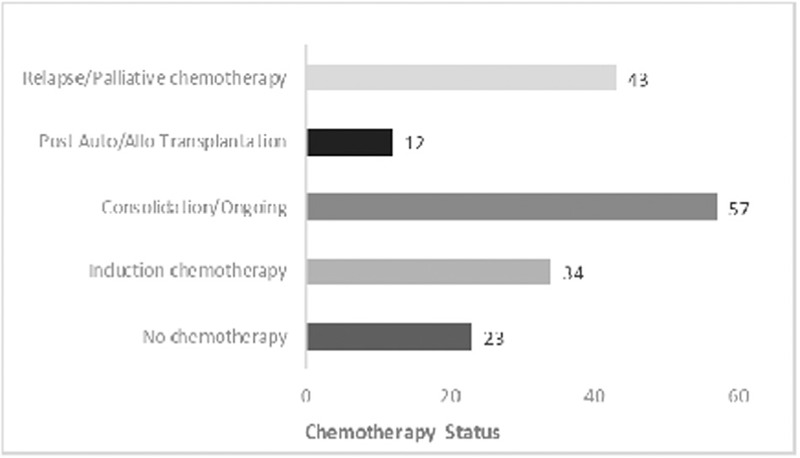
Chemotherapy status.

Patients were at various phases of their chemotherapy regimens, with most undergoing consolidation or other ongoing chemotherapy (*n* = 57). Only a minority of patients had undergone autologous or allogenic transplantation (*n* = 12). Chemotherapy status is outlined in Figure 3. One hundred eighteen (64%) patients were considered to be undergoing active treatment with a curative intention, while the remainder considered palliative. Based on their hematological risk profile, 65 patients (35.3%) were predicted to have an expected survival of less than 12 months.

### 3.4 Survival

Predicting for ICU survival outcome (as a binary variable) using simple logistic regression, we observed that a diagnosis of myeloma was also associated with ICU nonsurvival (*p* = 0.033). Induction chemotherapy (*p* < 0.0001) and posttransplantation therapy (*p* = 0.041) was predictive of ICU nonsurvival outcome but pre-chemotherapy and relapse treatment was not. The intent of chemotherapy, whether palliative or curative, was not predictive of ICU survival. APACHE scores were significant predictors of ICU nonsurvival outcome (*p* = 0.002 for APACHE II and *p* < 0.0001 for APACHE III).

A diagnosis of MDS and APACHE scores (*p* < 0.001) were similarly predictive for hospital admission nonsurvival for that episode as well. Induction (*p* = 0.002) and posttransplantation therapy (*p* = 0.009) was also associated with nonsurvival outcomes for hospital admission similar to ICU nonsurvival outcomes. A multivariate analysis for ICU or hospital admission survival outcome did not demonstrate significance for any variable.

Univariate analysis for overall survival in months (for a total follow-up period of 12 months) showed that superior ejection fractions (>55%) and prognosis (based on disease-specific risk scores) were significantly associated with overall survival (*p* = 0.024 and *p* = 0.001). On univariate analysis, age (*p* = 0.557), gender (*p* = 0.406), and hematological diagnosis (*p* = 0.135) were not associated with ICU outcomes. ECOG scores (*p* = 0.98) were also not significantly associated with overall survival. Furthermore, treatment status (*p* = 0.878) and intention of chemotherapy were also not statistically significant (*p* = 0.124). Multivariate analysis showed that ejection fraction and prognosis were independent predictors of overall survival.

**Figure 4:**
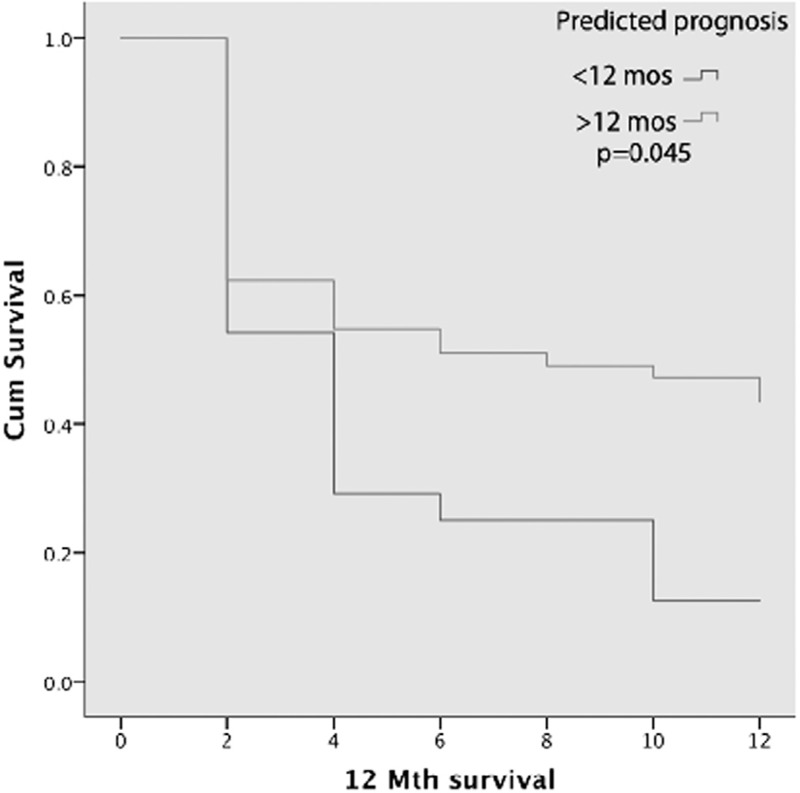
Kaplan–Meier curve showing significant overall survival difference.

The overall survival was higher in those with a pre-ICU disease-specific score that predicted for a >12 months life expectancy compared to those with a risk score that predicted for <12 months (*p* < 0.0001), as shown by Kaplan–Meir curve in shown in Figure 4.

## 4 DISCUSSION

This study aimed to identify the subgroups of HM patients that would benefit from an ICU admission. Better understanding the effect of non-acute illness factors on ICU survival can inform goals of care discussions early in the disease process, potentially preventing physical, psychological, and economic burden.^17^

While around one-third of patients benefited from ICU admission by surviving for a clinically significant period (at least 12 months), a similar proportion died within the ICU or in that hospital admission. The former outcome reinforces the role that intensive care has in the management of critically unwell patients with HM. Conversely, the latter outcome suggests a similar proportion of patients did not benefit from ICU admission, and it is important to understand why this may have occurred.

We evaluated a HM population across a broad range of ages, diagnoses, and treatments; the allogenic transplant group was underrepresented on account of the case mix in this center. Specifically, we found the plasma cell myeloma group had poorer outcomes in the univariate analysis for ICU survival. Previous studies have not consistently shown an adverse outcome for a specific HM and, in practice, a particular HM diagnosis would not exclude a patient from ICU admission.^9^ Furthermore, treatments for incurable HMs such as plasma cell myeloma have rapidly evolved in recent years with at least three new drugs now available in Australia changing the context of specific disease diagnosis in ICU referrals.^18^

Poor functional status, uncontrolled chronic comorbidities, and lack of reversibility in the underlying condition prompting referral are frequent reasons for ICU refusal in the general population.^4^ We found very few patients with a poor functional status and/or a large number of comorbidities, which may explain why ECOG and CCI scores did not reach significance. Pre-ICU ejection fraction, however, was a significant factor indicating the importance of cardiac function in ICU survival. This may also be a surrogate indicator for the overall low comorbidity status, good functional status, and younger age of the studied population.

For patients with a CCI score of 2—the minimum possible in this study—the expected 1-year mortality using original Charlson data is 26%, with worse outcomes for age and severity of illness.^19^ Our concern in using the CCI was that some malignancies may be given too much weight while others not enough.^20,21^ In certain HM such as AML, this can vary between months to years based on genotypic and phenotypic factors, while for some other HM, like DLBCL, the survival is uniformly greater than 1 year—which is not reflected in the CCI.^9^

APACHE scores, which are markers of critical illness severity, while not validated for the HM population, as expected showed significance for ICU survival outcomes. Based on the severity of their disease and/or critical illness, some patients were likely to have died in ICU. These patients may have been given an “ICU trial” in hope of a recovery, as a result of otherwise positive factors such as comorbidities, functional status, and cytogenetic profile.^22^

Acknowledging this heterogeneity of HM patients, we sought to determine individual prognosis based on HM-specific factors. We found that prognostication based on malignancy status, using disease-specific prognostic risk scores, was significantly associated with overall survival in both the univariate and multivariate analyses. These may be important to assist with ICU patient selection in the future.

The limitations of this study include a retrospective design and that genetic risk profiling was available for acute leukemia patients alone. Australia is fortunate in that it operates the ANZICS database that could be in future used to gather prospective multicenter information regarding patients with HM admitted to Australian ICUs.

The mortality rates described in this study are comparable to other contemporary Western data and also to the few Australian studies available.^21,23^ The high volume of HM admissions within this particular Australian ICU and the corresponding favorable outcomes of many of the patients reinforce the concept of a “specialized” ICU that is essential to improved outcome.^4^ This retrospective study is an important step in recognizing the changing contexts of ICU referrals in HM and planning prospective studies to understand the ICU outcomes for these patients.
